# Role of hepatocellular senescence in the development of hepatocellular carcinoma and the potential for therapeutic manipulation

**DOI:** 10.1007/s13577-025-01201-2

**Published:** 2025-03-18

**Authors:** Pramudi Wijayasiri, Stuart Astbury, Grace Needham, Philip Kaye, Mamatha Bhat, Anna M. Piccinini, Aloysious D. Aravinthan

**Affiliations:** 1 NIHR Nottingham Biomedical Research Centre, Nottingham University Hospitals NHS Trust and University of Nottingham, Nottingham, UK; 2 Nottingham Digestive Diseases Centre, Translational Medical Sciences, School of Medicine, University of Nottingham, E Floor, West Block, QMC Campus, Derby Road, Nottingham, NG7 2UH UK; 3 School of Pharmacy, University of Nottingham, Nottingham, UK; 4 Department of Pathology, Nottingham University Hospitals NHS Trust, Nottingham, UK; 5 Multiorgan Transplant Program, Toronto General Hospital, University of Toronto, Toronto, Canada

**Keywords:** Hepatocyte senescence, Hepatocellular carcinoma, Mammalian target of rapamycin, Senescence-associated secretory phenotype, Chemoprophylaxis

## Abstract

Accumulation of senescent hepatocytes is universal in chronic liver disease (CLD). This study investigates an association between hepatocyte senescence and hepatocellular carcinoma (HCC) and explores the therapeutic role of sirolimus. Background liver biopsies from 15 patients with cirrhosis and HCC and 45 patients with cirrhosis were stained for p16, a marker of cell senescence. STAM™ mice were randomized into 3 groups of 5 at 4 weeks of age and administered vehicle ± sirolimus intraperitoneally, thrice weekly, from 4 to 18 weeks of age. Placebo group was an administered vehicle, early sirolimus group was an administered vehicle with sirolimus, late sirolimus group was an administered vehicle from 4 to 12 weeks then vehicle with sirolimus from 12 to 18 weeks. The primary outcome was HCC nodule development. Senescent hepatocyte burden and senescence-associated secretory phenotype (SASP) factors were assessed in mice livers. In the human study, age (OR 1.282, 95% CI 1.086–1.513, *p* = 0.003) and p16 (OR 1.429, 95% CI 1.112–1.838, *p* = 0.005) were independently associated with HCC. In the animal study, all three groups exhibited similar MASLD activity scores (*p* = 0.39) and fibrosis area (*p* = 0.92). The number and the maximum diameter of HCC nodules were significantly lower in the early sirolimus group compared to placebo and late sirolimus group. The gene expression of SASP factors was similar in all groups. Protein levels of some SASP factors (TNFα, IL1β, IL-2, CXCL15) were significantly lower in sirolimus administered groups compared to placebo group. The study demonstrates an independent association between senescent hepatocyte burden and HCC. It indicates a potential chemoprophylactic role for sirolimus through SASP factor inhibition. These early results could inform a future human clinical trial.

**Graphical abstract:**

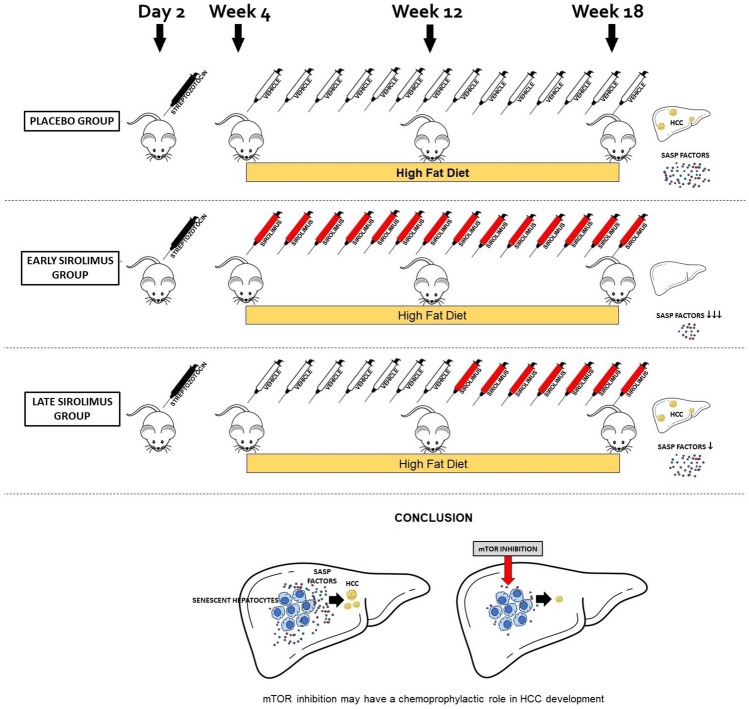

**Supplementary Information:**

The online version contains supplementary material available at 10.1007/s13577-025-01201-2.

## Introduction

The primary role of cellular senescence, a state of stable and permanent cell cycle arrest, is cancer prevention [1, 2]. However, cellular senescence has also been shown to play a role in tumorigenesis [3, 4] and hepatocyte senescence is no exception to this phenomenon. A murine model study elegantly illustrated hepatocellular carcinoma (HCC) development from senescent hepatocytes [5]. This is highly relevant to the development of HCC in advanced chronic liver disease (CLD) where accumulation of senescent hepatocytes appears to be a common characteristic irrespective of the liver disease etiology [6].

Escape of damaged hepatocytes from the senescent state is thought to be one of the two mechanisms involved in HCC development. While non-malignant hepatocytes in CLD expressed foci of DNA damage, shortened telomeres and cell cycle inhibitors indicating senescence, HCC from the same liver only expressed foci of DNA damage and shortened telomeres, without expressing cell cycle inhibitors [7], potentially indicating an escape from the senescent state. Gene expression in cirrhosis and HCC also demonstrates involvement of hepatocyte escape from senescence in HCC development [8]. The question of whether ongoing insult exacerbates DNA damage in already senescent hepatocytes, potentially enabling these cells to overcome replication barriers, remains unanswered.

Neighboring non-senescent hepatocytes in the vicinity of senescent hepatocytes may also give rise to tumors. Senescent cells, through senescence-associated secretory phenotype (SASP) factors, participate in angiogenesis, recruitment, and proliferation of new cells to replace damaged cells. This is a crucial role of senescent cells toward maintaining tissue integrity. However, this physiological phenomenon is suspected to be tumourigenic [9, 10]. The constituents of the SASP are diverse and vary depending on the context and cell type [3]. Several studies have highlighted the involvement of well-established SASP factors, including TNFα, IL1β, IL-6, IL8, VEGF, and various SASP-associated matrix metalloproteinases, in tumorigenesis [11–16]. The buildup of senescent hepatocytes in significant proportions as observed in advanced CLD creates a microenvironment abundant in SASP factors potentially fostering tumorigenesis in neighboring pre-senescent hepatocytes with DNA damage.

Mammalian target of rapamycin (mTOR) plays an essential role in the production of SASP factors, and inhibition of mTOR has been shown to downregulate SASP factors [17, 18]. Sirolimus, a potent mTOR inhibitor, has garnered significant interest in CLD research over the past two decades, primarily due to its remarkable anti-fibrotic properties [19–21]. A retrospective cohort study has also highlighted its potential role in preventing HCC [22]. Building on our current understanding of mTOR's role in SASP production and the involvement of SASP in tumorigenesis, it would be reasonable to hypothesize that mTOR inhibition could be used as chemoprophylaxis in CLD against the development of HCC. The purpose of this preliminary exploratory study was to investigate the role of mTOR inhibition in HCC development in a CLD model.

## Methods

### Liver biopsy specimens and histological quantification

Liver biopsy specimens were collected in accordance with guidelines established by the local research and ethics committee. Archived, formalin-fixed, paraffin-embedded needle biopsies from 15 patients with cirrhosis and hepatocellular carcinoma (HCC) were selected as case samples. These biopsies were taken from background liver tissue, distant from the HCC lesions. Archived, formalin-fixed paraffin-embedded biopsies from 45 etiology-matched patients with cirrhosis but without HCC served as controls.

Sections were stained for p16 (Roche, 1:200 concentration; heat-induced epitope retrieval (HIER) in Tris-based CC1 buffer, pH 8–8.5, at 95 °C for 64 min), a well-established marker of permanent cell cycle arrest and senescence [23]. This was used to assess the association between HCC and the burden of senescent hepatocytes. The proportion of senescent hepatocytes was quantified objectively by identifying p16-positive hepatocytes using FIJI software [24]. Whole-slide scans were captured using a NanoZoomer (Hamamatsu Photonics). Scripts from NDPItools [25] were employed to randomly extract three fields at 20× magnification from each slide. Extracted images were then deconvoluted using FIJI’s H-DAB setting, and nuclei were selected by thresholding on the blue channel, corresponding to the hematoxylin counterstain. p16-negative cells were counted automatically using the ‘analyze particles’ function, while p16-positive cells were manually counted and expressed as a percentage of the total cell population.

### STAM™ metabolic dysfunction-associated steatotic liver disease mouse model

Pathogen-free 14 day-pregnant C57BL/6 mice were obtained from Japan SLC, Inc. (Japan). The STAM™ model of metabolic dysfunction-associated steatotic liver disease (MASLD) was established in male mice through a single subcutaneous injection of 200 µg streptozotocin (Sigma, USA) administered 2 days post-birth, followed by ad libitum feeding of a high-fat diet (HFD; CLEA Japan Inc., Japan) starting at 4 weeks of age (day 28 ± 2). Mice were randomized into 3 groups of 5 at 4 weeks of age (day 28 ± 2), the day before the start of treatment. Placebo Group (STAM™ + Vehicle): 5 STAM™ mice were administered vehicle intraperitoneally in a volume of 5 ml/kg thrice weekly from 4 to 18 weeks of age. Early Sirolimus Group (STAM™ + Sirolimus): 5 STAM™ mice received intraperitoneal administration of vehicle supplemented with sirolimus at a dose of 1.5 mg/kg, three times per week, starting from 4 to 18 weeks of age. Late Sirolimus Group (STAM™ + Vehicle/Sirolimus): 5 STAM™ mice received intraperitoneal injections of vehicle at a volume of 5 ml/kg three times per week from 4 to 12 weeks of age, followed by intraperitoneal administration of vehicle supplemented with sirolimus at a dose of 1.5 mg/kg, also given three times weekly, from 12 to 18 weeks of age.

The treatment duration was determined based on the timeframe required for 100% of STAM mice to develop HCC, typically between 16 and 20 weeks of age [26]. The dose and the route of sirolimus administration were based on a prior study exploring sirolimus regimens in mice fed a high-fat diet. This study demonstrated the systemic effects of sirolimus in high-fat-fed mice without inducing significant metabolic abnormalities, using a dosage of 1.5 mg/kg administered intraperitoneally three times per week [27].

The in-life phase of the animal study was conducted at SMC Laboratories, Inc. Tokyo, Japan, a contract research organization. SMC Laboratories operates under the oversight of the Ministry of Education, Science and Technology, Japan, is regularly audited on animal welfare standards by global pharmaceutical companies, and possesses the necessary ethical approvals to conduct animal research (SLMN009-1704-0). All mice were kept in a specific pathogen-free environment within TPX™ cages (CLEA, Japan), where conditions were carefully regulated, including temperature (23 ± 2 °C), humidity (45 ± 10%), and a 12-h light/dark cycle (lights on from 08:00 to 20:00). The facility also maintained controlled air exchange and negative pressure in the experimental room to prevent contamination. Sterilized solid HFD was provided ad libitum, placed in a metal lid on top of the cage. Fresh water was freely available via a water bottle fitted with a rubber stopper and a sipper tube. The water bottles were replaced weekly, cleaned, sterilized in an autoclave, and reused. Sterilized Paper-Clean (Japan SLC) was used as bedding and was changed once a week. Viability, clinical signs, and behavior of the mice were monitored daily. Body weight was measured prior to treatment. Following each administration, the mice were observed for 60 min to check for signs of toxicity, moribundity, and mortality.

All mice were sacrificed at 18 weeks of age by exsanguination through direct cardiac puncture under isoflurane anesthesia (Pfizer Inc.). The extirpated livers were examined macroscopically for the presence of gross swelling tumor nodules and tumor nodules > 2 mm in diameter were counted. Two sections from the left lateral lobe, as well as from the left and right medial and caudate lobes, were snap-frozen in liquid nitrogen and stored at − 80 °C. The remaining portions were fixed in Bouin’s solution, embedded in paraffin, and kept at room temperature. Sections from paraffin blocks of liver tissue were cut for hematoxylin and eosin staining to assess MASLD activity score and picrosirius red staining to assess fibrosis. Fibrosis area was quantitatively assessed by capturing bright-field images of picrosirius red-stained sections around the central vein with a digital camera (DFC295; Leica, Germany) at 200× magnification. The positive areas in five fields per section were measured using ImageJ software (National Institute of Health, USA). All slides were reviewed by an independent liver histopathologist (PK).

Non-fasting blood samples were collected in separate serum tubes (BD, USA) without anticoagulant and centrifuged at 15,000×*g* for 2 min at 4 °C. The supernatant was then collected and stored at − 80 °C. Serum alanine aminotransferase (ALT) levels were measured using the FUJI DRI-CHEM 7000 (Fujifilm Corporation, Japan). Total lipid extracts from the liver were obtained using Folch’s method [28]. Liver samples were homogenized in chloroform–methanol (2:1 V/V) and incubated overnight at room temperature. After washing with chloroform–methanol–water (8:4:3 V/V/V), the extracts were evaporated to dryness and dissolved in isopropanol. Liver triglyceride content was determined using the Triglyceride E-Test (Wako Pure Chemical Industries Ltd., Japan).

### Liver tissue preparation and quantification of SASP factors

Frozen liver lobes from STAM™ mice were pulverized using a Cryo-Cup Grinder (Biospec, USA) following manufacturer’s instructions. Tissue lysis was conducted by re-suspending pulverized tissue in ice-cold T-PER tissue protein extraction reagent (Thermo Fisher Scientific) containing protease inhibitor cocktail (working dilution 1:1000; Roche), at a final concentration of 100 mg/ml. Following sonication on ice (80 s total time; pulse ON for 10 s; pulse OFF for 30 s; amplitude 40%), samples were centrifuged at 10,000 rpm for 5 min at 4 °C. The protein concentration of each sample in the supernatant was measured using a Coomassie (Bradford; Bio-Rad) protein assay. Supernatants were then analyzed for the presence of the following SASP factors: TNF-α, IL-1β, and IL-6 by enzyme-linked immunosorbent assay (ELISA) following manufacturer’s instructions (R&D Systems). A microplate reader (BioTek Synergy HTX) read the absorbance at 450 nm and analyzed using the four parameter logistic (4PL) regression model.

For western blotting analysis, liver protein extracts were separated on 14% SDS-PAGE gels, and proteins were transferred to nitrocellulose membranes. The membranes were blocked in 5% bovine serum albumin (BSA, Merck) in tris-buffered saline (TBS) containing 0.1% Tween 20 (TBST) and were sequentially incubated with antibodies against mouse CXCL15 (bs-2554R; Bioss Antibodies), IL-2 (MAB402; R&D Systems), α-tubulin (ab52866; Abcam), and β-actin (4970S; Cell Signaling Technology). Both α-tubulin and β-actin were used as housekeeping proteins in the western blot experiments as their expression is not affected by sirolimus [29–31]. Blots were stripped of antibody between analyses with ReBlot Plus Strong Antibody Stripping Solution (Merck Millipore) and blocked again in 5% BSA-TBST. Densitometric analysis of bands was performed with Fiji, and the results are presented as relative band volumes.

### RNA extraction and QT-PCR

RNA was extracted from mouse livers using phenol/chloroform separation and the Qiagen RNeasy Mini Kit (#74104). Extracted RNA was quantified using a Nanodrop before normalization to 500 ng total RNA and reverse transcription using the High-Capacity RNA to cDNA kit (Thermo Fisher #4387406). TaqMan probes were used for qPCR (TaqMan Fast Advanced Master Mix, Thermo Fisher #4444964). The expression of all genes was adjusted to that of GAPDH, and their relative expression calculated using the 2^ΔΔCt^ method. Probes used and Thermo Fisher assay IDs are as follows: GAPDH (Mm99999915_g1); TNFα (Mm00443258_m1); IL1β (Mm00434228_m1); and IL-6 (Mm00446190_m1).

### Statistical analysis

Statistical analysis was undertaken using GraphPad prism 10 and SPSS (version 28). *p* < 0.05 was considered significant. Results were expressed as mean with standard deviation (SD) or median with interquartile range (IQR) or number with percentage unless otherwise stated. All statistical analyses were performed using either GraphPad prism 10 (San Diego, CA) or SPSS for Windows v28 (IBM Corp, Armonk, NY, USA). Clinical parameters at biopsy were analyzed for association with the development of HCC using the Mann–Whitney *U* test or 1-way ANOVA (Kruskal–Wallis test) as appropriate. A multivariable binary logistic regression model was employed to identify independent associations with the development of HCC, by including variables that had a *p* value of < 0.10 in univariate analysis. Variables were only considered to have independent association if the *p* value reached Bonferroni-corrected level of significance.

## Results

### Association between hepatocyte senescence and hepatocellular carcinoma

**Fig. 1:**
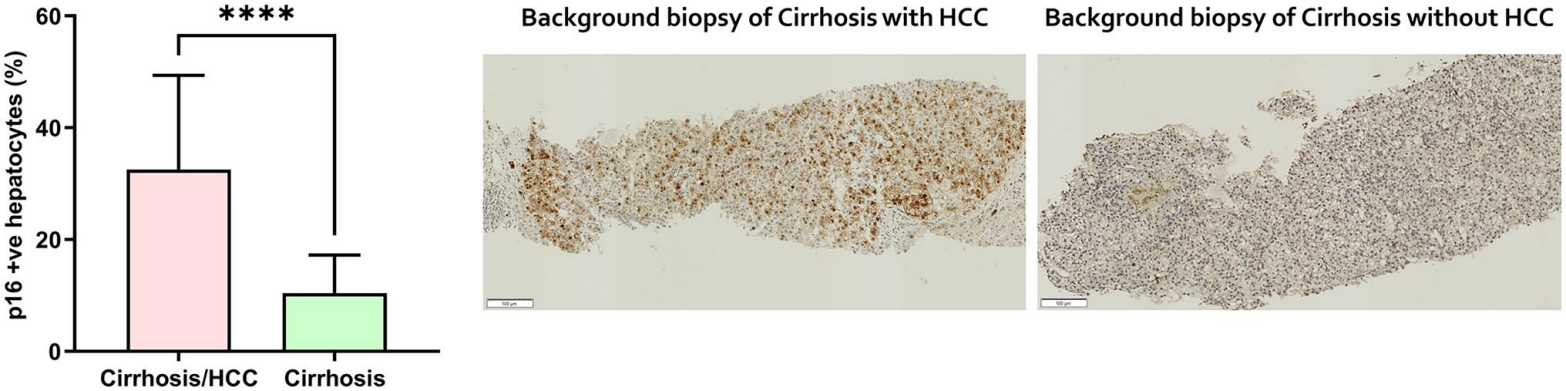
Hepatocyte senescence in the background human livers of patients with cirrhosis and cirrhosis with HCC. The figure illustrates a significantly higher proportion of senescent hepatocytes, as indicated by p16-positive hepatocytes, in the background human livers of patients with cirrhosis and hepatocellular carcinoma compared to patients with cirrhosis alone. Data are presented as means ± SD. Patients with cirrhosis and HCC *n* = 15; patients with cirrhosis and without HCC *n* = 45. ****p < 0.0001 by Mann–Whitney test

The demographic and clinical characteristics of 15 patients with cirrhosis and HCC and etiology-matched 45 patients with cirrhosis alone are summarized in Table 1. On univariate analysis, older age of the patient, lower serum albumin, lower platelet count, and higher expression of p16 in hepatocytes were associated with the development of HCC. The expression of hepatocyte p16 in the background liver tissue was significantly higher in patients with cirrhosis and HCC compared to patients with cirrhosis alone [median 27.7% (IQR 22.6–42.9) vs. median 9.5% (IQR 4.1–17.4), *p* < 0.0001; Fig. 1]. On multivariate analysis (Table 1), age of the patient [OR 1.282 (95% CI 1.086–1.513), *p* = 0.003] and hepatocyte expression of p16 [OR 1.429 (95% CI 1.112–1.838), *p* = 0.005] were the only independent predictors of HCC development in cirrhosis, even after the Bonferroni-corrected level of significance.

**Table 1:** Demographic and clinical parameters of patients with cirrhosis and HCC and cirrhosis alone

	Cirrhosis/HCC (*n* = 15) Median (IQR) or number (%)	Cirrhosis (n = 45) Median (IQR) or number (%)	*p* value (univariate)	OR (95% CI)	*p* value (multivariate)
Age at biopsy (years)*	71.9 (63.2–77.0)	56.3 (47.8–62.5)	< 0.0001*	1.282 (1.086–1.513)	0.003
Male sex	11 (73%)	26 (57.8%)	0.44		
BMI at biopsy	33.0 (29.0–37.0)	32.0 (27.0–34.5)	0.52		
Etiology					
ALD	5 (33.3%)	19 (42.2%)	0.81		
MASLD	8 (53.3%)	22 (48.9%)			
HCV/HBV	1 (6.7%)	1 (2.2%)			
Autoimmune liver disease	0 (0.0%)	1 (2.2%)			
Others	1 (6.7%)	2 (4.4%)			
Bloods at biopsy					
ALT (IU/l)	39 (22–71)	46 (24–71)	0.47		
AST (IU/l)	41 (35–79)	60 (36–79)	0.51		
AST/ALT Ratio	1.3 (0.9–1.7)	1.6 (0.9–2.0)	0.26		
ALP (U/l)*	142 (107–217)	117 (84–191)	0.08*	1.005 (0.994–1.016)	0.37
Bilirubin (μmol/l)	17 (12–24)	14 (9–23)	0.17		
Albumin (g/l)*	36 (29–42)	41 (39–42)	0.04*	1.062 (0.867–1.302)	0.56
PT (s)	12 (12–13)	12 (11–13)	0.26		
Platelets (× 10^9^)*	120 (83–163)	161 (129–238)	0.04*	0.972 (0.942–1.003)	0.07
HbA1C	49 (33–59)	45 (33–61)	0.98		
HCC					
Number of lesions	1 (1–2)				
Maximum diameter (mm)	20 (28–43)	na	na		
Hepatocyte p16 expression*	27.7 (22.6–42.9)	9.5 (4.1–17.4)	< 0.0001*	1.429 (1.112–1.838)	0.005

Parameters with a *p* value < 0.10 on univariate analysis were included in the multivariate analysis and these parameters are indicated by an asterisk (*). The Bonferroni-corrected level of significance in this analysis was *p* < 0.01 and is indicated in bold

*ALD* alcohol-related liver disease, *ALP* alkaline phosphatase, *ALT* alanine aminotransferase, *AST* aspartate aminotransferase, *BMI* body mass index, *HbA1C* glycated haemoglobin, *HBV* hepatitis B-related liver disease, *HCV* hepatitis C-related liver disease, *MASLD* metabolic dysfunction-associated steatotic liver disease, *PT* prothrombin time

### Sirolimus and STAM™ mice—general features

**Fig. 2:**
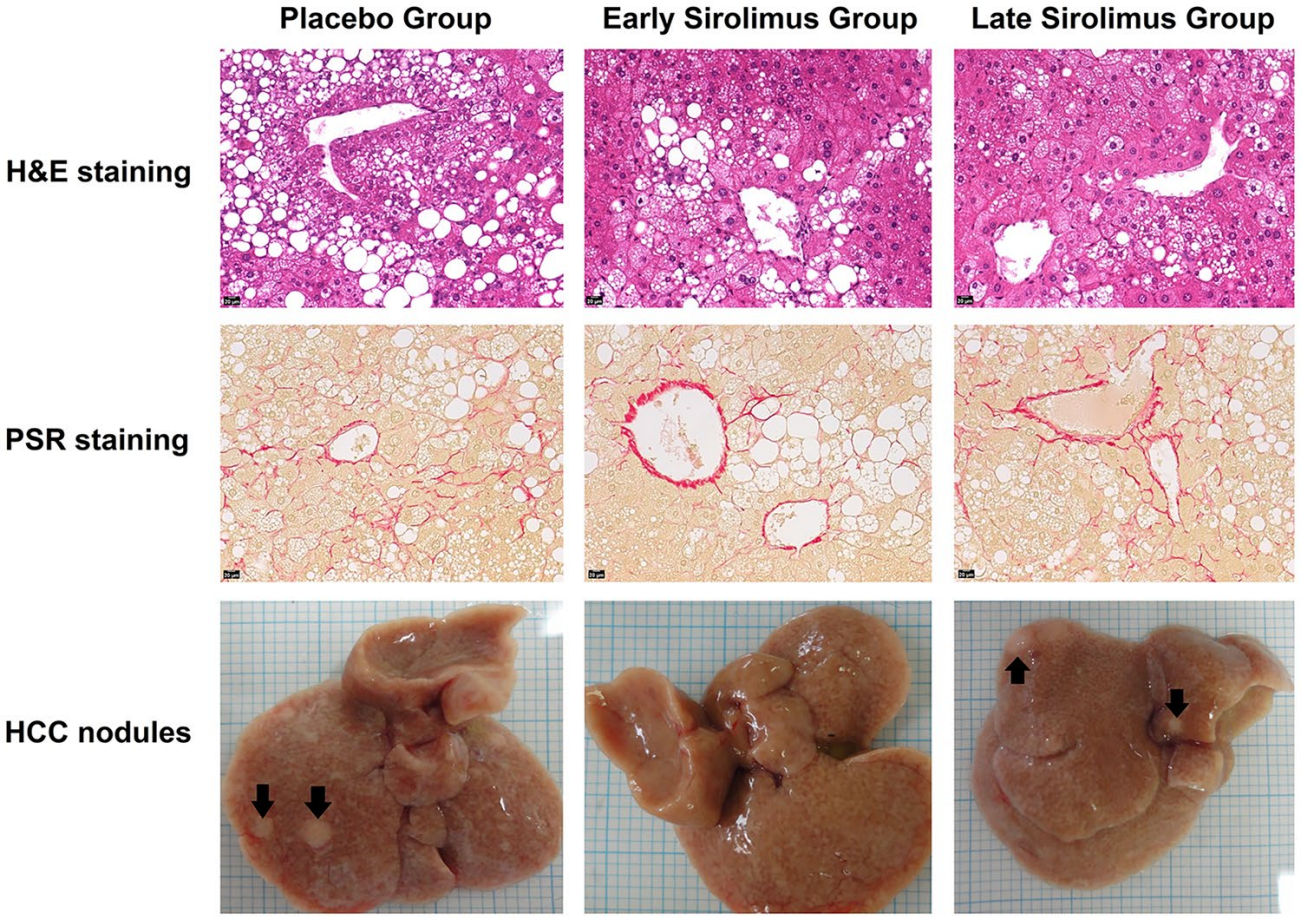
Steatosis, fibrosis and HCC nodules in Placebo Group, Early Sirolimus Group and Late Sirolimus Group mice. Representative H&E stained, picrosirius red-stained and gross morphology of liver with hepatocellular carcinoma nodules of placebo, early sirolimus and late sirolimus mice indicate no significant difference in steatosis and fibrosis between the groups. However, the number of hepatocellular nodules was significantly lower in early sirolimus group compared to the other two groups. *n* = 5 mice per group

The findings of the three groups of mice (Placebo, Early Sirolimus and Late Sirolimus) are summarized in Table 2. Mean body weight at week 18 of Early Sirolimus Group mice was significantly lower than the other two groups (15.9 ± 2.8 g vs. Placebo Group 21.6 ± 2.1 g; *p* = 0.006 and Late Sirolimus Group 20.3 ± 1.0 g; *p* = 0.01). However, there was no difference in mean body weight at week 18 between Placebo Group mice and Late Sirolimus Group mice (*p* = 0.25). There were no differences in liver weight or spleen length (major axis and minor axis) between the three groups (Table 2). Although the serum ALT and liver triglyceride content were numerically higher in Early Sirolimus Group mice compared to the other two groups, it did not reach statistical significance (Table 2). Liver sections from all three groups exhibited micro- and macrovesicular steatosis, hepatocellular ballooning and inflammatory cell infiltration. There was no significant difference in MASLD activity score (one-way ANOVA *p* = 0.39) between the three groups (Table 2; Fig. 2). All three groups exhibited early stage fibrosis (F1), and the fibrosis areas, represented by picrosirius red-positive area, were similar in all three groups (one-way ANOVA *p* = 0.92) (Table 2; Fig. 2).

**Table 2:** General and clinical parameters of STAM™ mice at the end of experiment (week 18)

	Placebo Group (STAM™ + Vehicle) Mean ± SD	*p* value Early Sirolimus Group vs. Placebo Group	Early Sirolimus Group (STAM™ + Sirolimus) Mean ± SD	*p* value Early Sirolimus Group vs. Late Sirolimus Group	Late Sirolimus Group (STAM™ + Vehicle/Sirolimus) Mean ± SD	*p* value Late Sirolimus Group vs. Placebo Group
Body weight (g)	21.6 ± 2.1	**0.006**	15.9 ± 2.8	**0.01**	20.3 ± 1.0	0.25
Liver weight (mg)	1738 ± 261	0.46	1621 ± 213	0.10	1855 ± 191	0.44
Spleen major axis (mm)	17 ± 5	0.22	14 ± 1	0.15	15 ± 1	0.99
Spleen minor axis (mm)	6 ± 1	0.15	5 ± 1	1.0	5 ± 1	0.15
Serum ALT (U/L)	65 ± 19	0.10	153 ± 104	0.09	64 ± 12	0.92
Liver triglyceride (mg/g)	53.4 ± 23.4	0.07	86.4 ± 26.7	0.08	60.3 ± 11.1	0.57
MASLD Activity Score	4.4 ± 0.8	0.84	4.3 ± 0.8	0.27	3.8 ± 0.5	0.19
Fibrosis area	0.90 ± 0.21	0.32	1.01 ± 0.22	0.25	0.88 ± 0.19	0.25
Macroscopic HCC nodules	1.3 ± 1.0	**0.02**	0.0 ± 0.0	**0.02**	1.5 ± 1.2	0.78
Maximum diameter of HCC	2.9 ± 1.7	**0.005**	0.0 ± 0.0	**0.02**	2.8 ± 2.2	0.94

Values in bold indicate statistically significant *p*-values (*p* < 0.05)

Placebo Group (STAM™ + Vehicle)—5 STAM™ MASLD mice were administered vehicle intraperitoneally in a volume of 5 ml/kg thrice weekly from 4 to 18 weeks of age; Early Sirolimus Group (STAM™ + Sirolimus)—5 STAM™ MASLD mice were administered vehicle supplemented with Sirolimus intraperitoneally at a dose of 1.5 mg/kg thrice weekly from 4 to 18 weeks of age; Late Sirolimus Group (STAM™ + Vehicle/Sirolimus)—5 STAM™ MASLD mice were administered vehicle intraperitoneally in a volume of 5 ml/kg thrice weekly from 4 to 12 weeks of age, followed by vehicle supplemented with Sirolimus intraperitoneally at a dose of 1.5 mg/kg thrice weekly from 12 to 18 weeks of age

### Sirolimus and STAM™ mice—hepatocellular carcinoma

The number of HCC nodules was significantly higher in Placebo Group mice (*p* = 0.02) and Late Sirolimus Group mice (*p* = 0.02) compared to Early Sirolimus Group mice (Table 2; Fig. 2). Similarly, the maximum diameter of HCC nodule was significantly larger in Placebo Group mice (*p* = 0.005) and Late Sirolimus Group (*p* = 0.02) mice compared to Early Sirolimus Group mice. However, there were no differences in the number (*p* = 0.78) or the maximum diameter (*p* = 0.94) of HCC nodules between Placebo Group mice and Late Sirolimus Group mice.

### Sirolimus and STAM™ mice—hepatocyte senescence and SASP factors

**Fig. 3:**
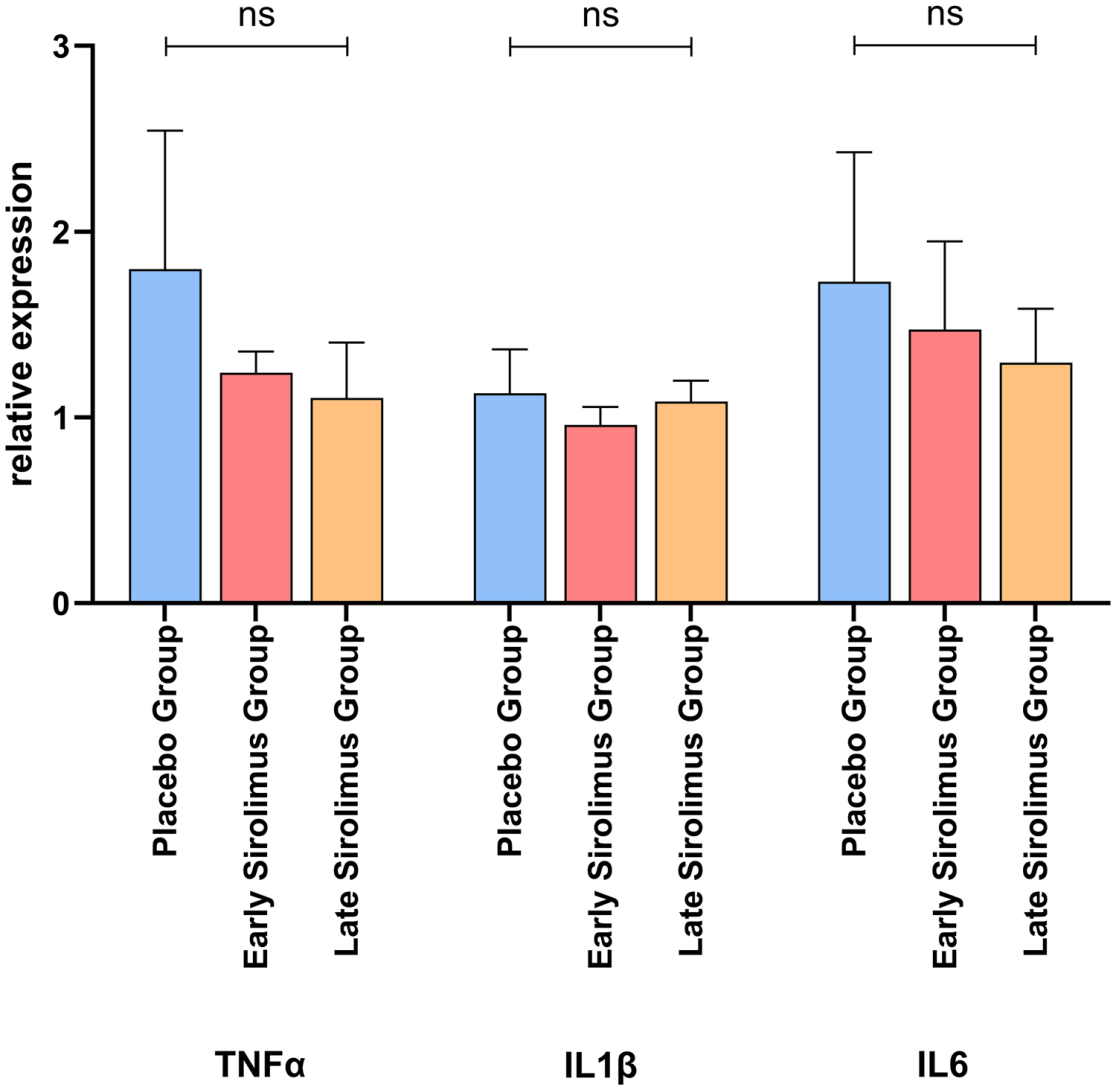
Gene expression of senescence-associated secretory phenotype (SASP) factors in Placebo Group, Early Sirolimus Group and Late Sirolimus Group mice. There were no differences in the gene expression of SASP factors TNFα, IL1β and IL-6 in the three groups. Data are presented as means ± SD. *n* = 5 mice per group. Statistically non-significant (ns) *p* by one-way ANOVA

**Fig. 4:**
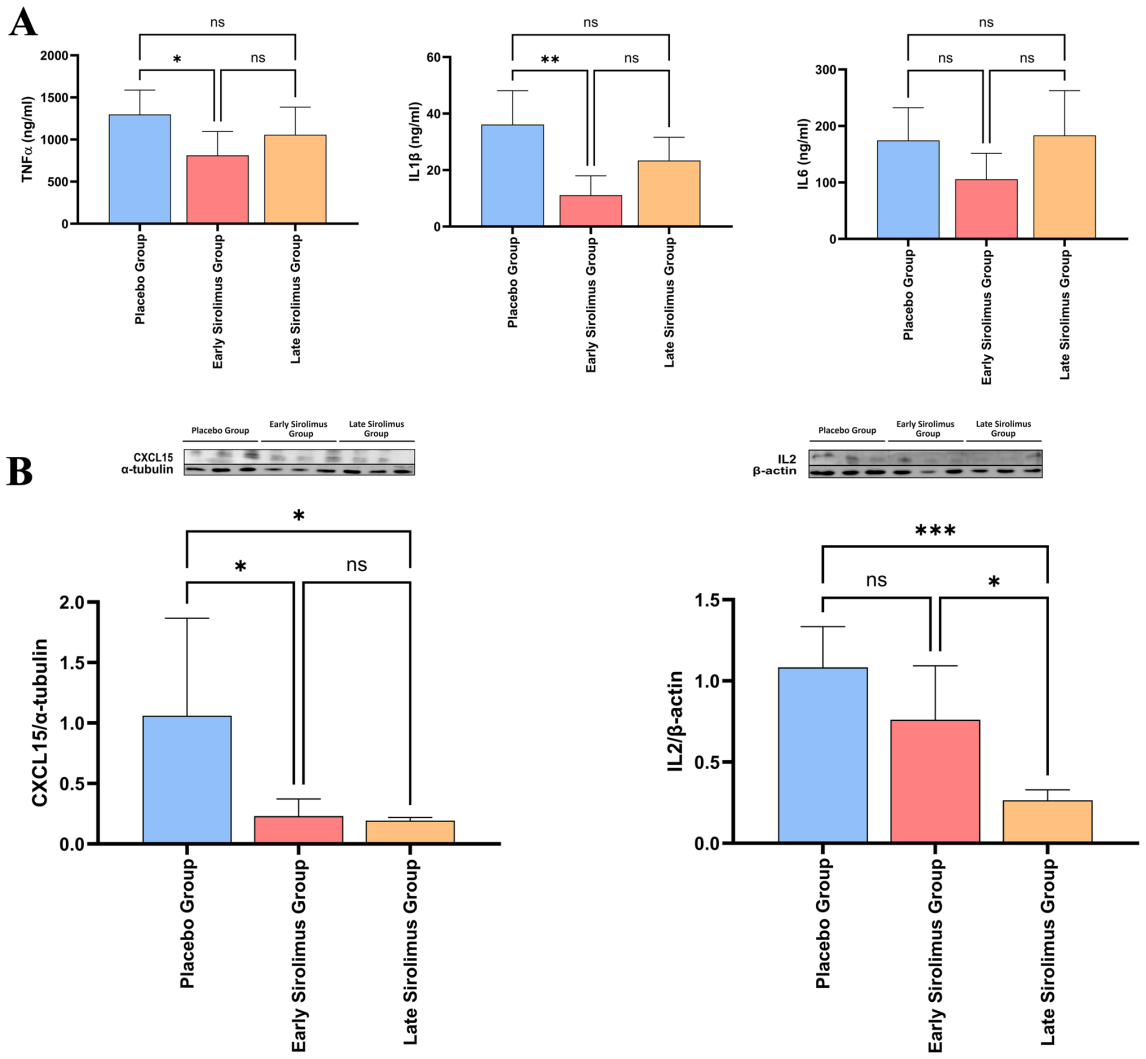
Analysis of senescence-associated secretory phenotype (SASP) factors in the livers of STAM™ mice. **A** The amounts of TNF-α, IL-1β and IL-6 present in the liver tissue lysates of Placebo Group, Early Sirolimus Group and Late Sirolimus Group were quantified by ELISA. Data is presented as means ± SD. **B** Top: Liver tissue lysates were subjected to western blotting analysis with antibodies specific for IL-2, CXCL15, β-actin and α-tubulin. Bottom: Histograms show quantification of the abundances of IL-2 and CXCL15 normalized to that of β-actin and α-tubulin, respectively, by densitometric analysis. Data are presented as means ± SD. *n* = 5 mice per group. *p < 0.05, **p < 0.01, and ***p < 0.001 by one-way ANOVA

The hepatic gene expressions of TNFα (*p* = 0.65), IL1β (*p* = 0.79) and IL-6 (*p* = 0.88) were similar in all three mice groups (Fig. 3). At the protein level, all SASP factors analyzed were present but in lower amounts in the liver of Early Sirolimus Group mice compared to Placebo Group mice, with TNFα (*p* = 0.039), IL1β (*p* = 0.002) and CXCL15 (*p* = 0.041) reaching statistical significance and IL-2 (*p* = 0.139) and IL-6 (*p* = 0.241) being non-significant. Further, the livers of Late Sirolimus Group mice contained significantly lower levels of IL-2 (*p* = 0.0008) and CXCL15 (*p* = 0.0384) compared to Placebo Group mice. However, though the levels of TNFα (*p* = 0.457) and IL1β (*p* = 0.1915) were lower in Late Sirolimus Group mice than those in Placebo Group mice, they did not reach statistical significance, and the amount of IL-6 (*p* = 0.2213) in Late Sirolimus Group mice was like that of Placebo Group mice (Fig. 4a, b).

## Discussion

This preliminary exploratory study investigates the association between hepatocyte senescence, mTOR inhibition and HCC development in CLD. It demonstrates an independent association between hepatocyte senescence, as reflected by p16 expression, and the development of HCC in patients with cirrhosis. Further, the study suggests that early mTOR inhibition may suppress HCC development in CLD.

The primary function of cellular senescence is cancer prevention [32]. Cellular senescence induces permanent cell cycle arrest in cells that have acquired genomic instability through cellular damage and are at risk of becoming cancerous [33, 34]. However, cellular senescence has also been shown to play a paradoxical role in tumor promotion. SASP factors, which promote inflammation, induce senescence in nearby damaged cells and eventually facilitate the removal of senescent cells [35], have also been shown to fuel proliferation of pre-malignant cells and malignant transformation [9, 10]. The latter has been shown to occur when senescent cells are allowed to persist and enter a deep senescent state where they acquire phenotypic diversification and become detrimental to the tissue [5, 36]. Several individual SASP factors have been identified in previous studies, with different factors appearing to influence distinct aspects of cancer development including cancer cell differentiation, proliferation, invasion, and immune evasion [4]. Disruption of breast epithelial cell differentiation occurs in the presence of senescent conditioned media, mediated by matrix metalloproteinases (MMPs) [10]. Similarly, MMP-driven permeability of local capillaries has been shown to promote the growth of neighboring co-transplanted cancer cells [16]. Senescent cells have been shown to generate a SASP factor gradient that facilitates tumor cell migration and invasion [4]. In a previous study, TNFα was found to promote HCC cell proliferation and migration [37]. IL-6 and IL8, secreted by senescent fibroblasts in breast cancer, drive epithelial-to-mesenchymal transition in cancer cells and enhance the invasiveness of multiple cultured cancer cell lines [38]. VEGF induces endothelial cell migration and invasion, a process intensified in tissues containing senescent cells [39]. IL1β increases endothelial cell adhesiveness, enabling cancer cells to adhere to vessel walls [40, 41]. While IL-6 is closely associated with HCC development [42], its inhibition has paradoxically been shown to promote HCC progression in a CLD mouse model [43]. CXCL15 recruits macrophages to HCC tumor margins, suppressing the anti-tumor immune response and promoting invasion [44]. The sheer diversity of SASP factors, their combinations, and the contexts in which they operate highlight the significant gaps in our understanding of their causal links.

In the human cohort, higher p16 expression in hepatocytes, a marker of hepatocyte senescence, independently predicted HCC development. This is consistent with the theory that senescent cells, although initially tumor-suppressive, may contribute to tumorigenesis in a chronically damaged environment by creating a pro-angiogenic and pro-tumourigenic milieu [9, 16, 45, 46]. This underscores the significance of senescent hepatocyte accumulation in the pathogenesis of HCC development in CLD, an association demonstrated in previous studies [5, 8, 47]. Further, cell senescence has also been implicated in cancers of other organs [48–51], reinforcing the detrimental effect of persistence of senescent cells in tumorigenesis. The opposing effects of cell senescence on tumorigenesis have been shown to be due to variations in SASP factors under different circumstances [52, 53].

The animal study using STAM™ mice offered further insights into the association between hepatocyte senescence and HCC development. Sirolimus, a potent mTOR inhibitor, effectively reduced the development and growth of HCC nodules when administered early. This suggests that mTOR plays a crucial role in the development of HCC, potentially through regulating the SASP factors. Despite no significant differences in liver weight, spleen length, liver triglyceride content, level of steatohepatitis, fibrosis or serum liver biochemistry among the groups, the reduced number and the size of HCC nodules in the early sirolimus-treated group point toward its potential as a chemoprophylactic agent. Inhibition of mTOR by sirolimus did not lead to a significant reduction in the gene expression of TNFα, IL1β, IL-6, but suppressed these SASP factors at a protein level, likely through inhibition of mRNA translation, a well-described mTOR mediated function in protein synthesis [54]. This notion is supported by previous studies demonstrating the crucial role of mTOR in SASP production, and mTOR inhibition suppressing the senescent cells’ ability to promote tumor growth in mice [17, 18]. A study of liver transplant recipients demonstrating a significantly prolonged median time to HCC recurrence in those who received sirolimus-based immunosuppression [22] further corroborates the findings of our study.

In vitro studies show an anti-fibrotic effect with sirolimus treatment [55]. Sirolimus has been shown to reduce fibrosis even after the development of cirrhosis in animal models [19, 21]. In humans, a retrospective analysis of liver transplant recipients demonstrated a significant reduction in the rate of fibrosis progression with sirolimus [22]. However, in the current study, there was no difference in fibrosis among the STAM™ mice groups. One possible explanation for this is that the degree of fibrosis was not enough to appreciate a meaningful difference.

It is imperative to recognize the limitations in our study. The small sample size and the use of a specific animal model may limit the generalizability of the findings. The STAM™ mouse model is an established model for research of steatohepatitis to HCC progression, but, unlike in most human CLD, the model does not develop advanced fibrosis/cirrhosis before developing HCC [56]. Consequently, there will be differences in the pathogenesis of HCC in STAM™ mice compared to the conventional human pathway. Nonetheless, it's noteworthy that fatty liver stands as a prominent precursor to HCC, with approximately 20% of cases occurring independent of underlying cirrhosis [57]. Further work with this model may prove useful to understand this phenomenon. While the expression of p16, a marker indicating permanent cell cycle arrest, is a well-established characteristic of cellular senescence [58], the absence of a universally accepted biomarker for detecting senescent cells both in vitro and in vivo presents a limitation. Additionally, while the association between senescence and HCC development was evident, the exact mechanisms and the roles of SASP factors remain to be fully elucidated. Future research should focus on a larger cohort and delve deeper into the molecular pathways linking senescence to HCC.

In conclusion, this study highlights the dual role of hepatocyte senescence in HCC development and underscores the potential of mTOR inhibition as a strategy to mitigate the risk of HCC in patients with CLD. This could open new avenues for the prevention and treatment of HCC, a significant complication of CLD.

## Supplementary Information

Below is the link to the electronic supplementary material.

Supplementary file1 (PDF 838 KB)

## Data Availability

Data is available upon request from the corresponding author.
