# Supplementary figures and images for: Role of hepatocellular senescence in the development of hepatocellular carcinoma and the potential for therapeutic manipulation
Source: Hum Cell. 2025 Mar 18;38(3):70. doi: 10.1007/s13577-025-01201-2 (PMC11920335; doi:10.1007/s13577-025-01201-2)

$\beta$ -ACTIN

45kDa

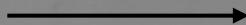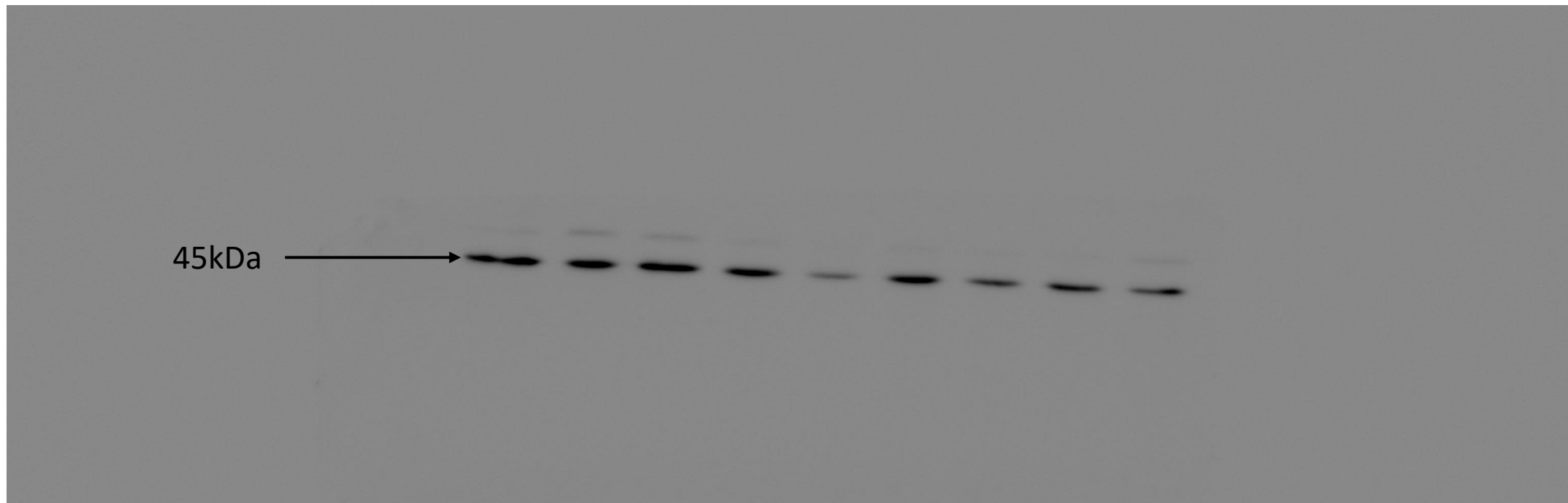

# $\alpha$ -TUBULIN

50kDa

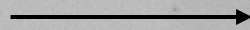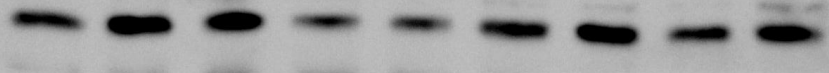

# CXCL15

18kDa →

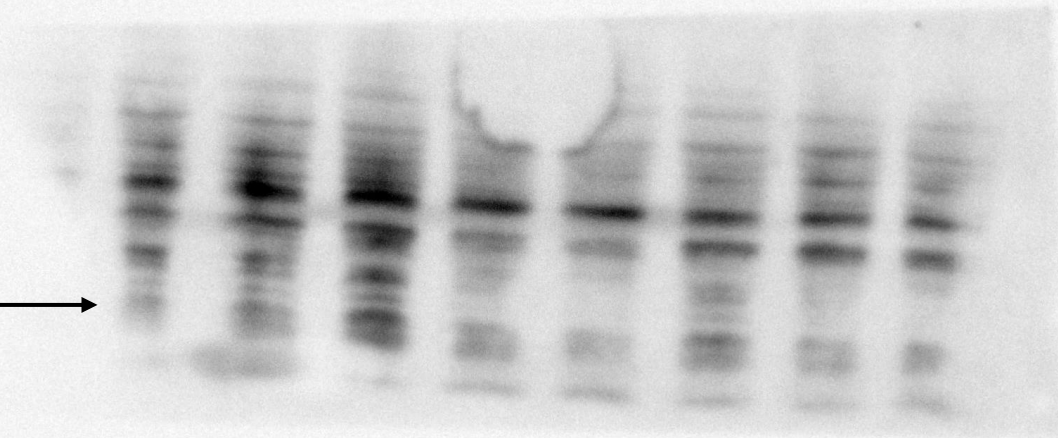

IL2

16kDa →

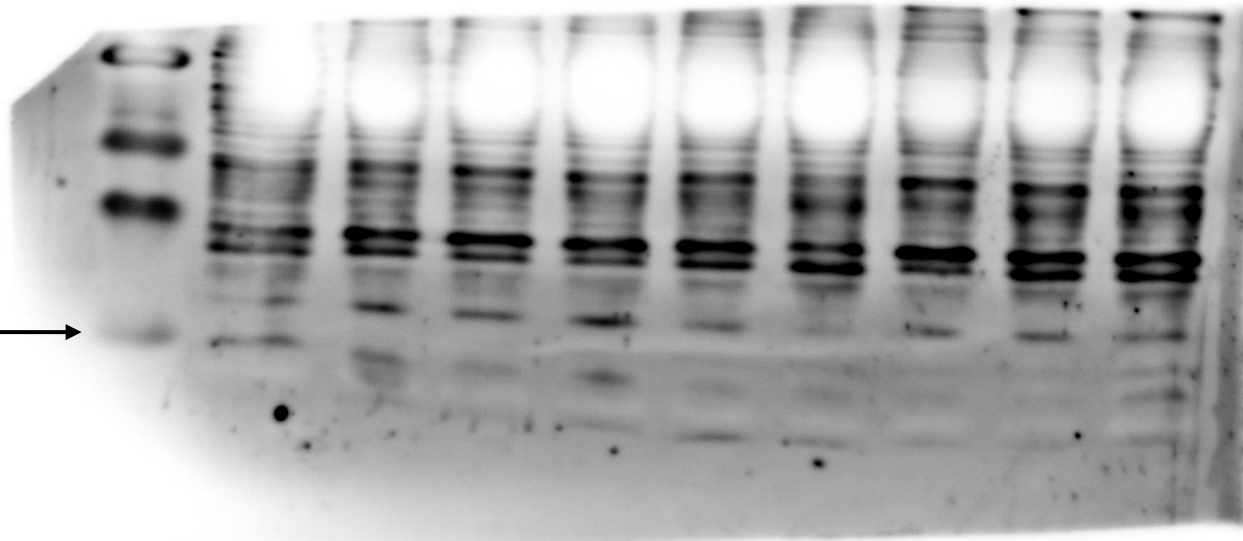

Supplement: Supplementary file 1 — Supplementary file1 (PDF 838 KB) [file 13577_2025_1201_MOESM1_ESM.pdf]
